# Nexus of Ethical Leadership, Career Satisfaction, Job Embeddedness, and Work Engagement in Hospitality Industry: A Sequential Mediation Assessment

**DOI:** 10.3389/fpsyg.2022.865899

**Published:** 2022-05-20

**Authors:** Shoukat Iqbal Khattak, Aftab Haider, Syed Khalil Ahmed, Syed Tahir Hussain Rizvi, Lin Shaokang

**Affiliations:** ^1^School of Business Administration, Jimei University, Xiamen, China; ^2^Business Studies Department, Bahria University, Islamabad, Pakistan; ^3^Department of Management Sciences, University of Loralai, Balochistan, Pakistan; ^4^Department of Management Sciences, Faculty of Management Sciences, International Islamic University, Islamabad, Pakistan; ^5^School of Finance, Renmin University of China, Beijing, China

**Keywords:** ethical leadership, job embeddedness, work engagement, career satisfaction, hospitality industry

## Abstract

The paper proposes a research model explaining the sequential mediation effect of job embeddedness (JE) and work engagement (WENG) between ethical leadership (EL) and career satisfaction (CS). The model also examines whether JE heightens WENG, a factor indirectly influenced by ethical practices ending in employee satisfaction. The study used a time-lagged data collection procedure and survey responses of 247 hotel workers in China. Data were analyzed through structural equation modeling. The results showed that EL directly and indirectly (through sequential mediation effect of JE and WENG) contributes to employee CS. The present empirical framework extends the hospitality industry literature by explaining the precise mechanism (i.e., JE and WENG) through which EL generates CS among hospitality workers in China. The paper offers theoretical and practical implications and future research directions.

## Introduction

The growth and sustainability of modern enterprises, especially in the current volatile and dynamic era, are considered impractical without the engagement and keenness of the workforce (Boğan et al., 2020). Many experts believe that the success and prosperity of enterprises depend upon employee willingness to extend beyond specific technical and contracted tasks and their readiness to participate in extra-role activities voluntarily (Harari et al., 2016). Globalization and competition make it difficult for modern managers to enhance cooperation among organizational members (Joo and Nam, 2019). Financial compensations (e.g., pay and perks) are now perceived as insufficient resources to retain the support of organizational members (Asante Boadi et al., 2020). Past empirical evidence supports that factors, including ethical climate, trustworthiness, respect, and care for employee emotions and sentiments, career satisfaction (CS), job embeddedness (JE), work engagement (WENG), corporate social responsibility, organizational prestige, pride, and leadership style, play a significant role in determining employee behavior and attitude toward the organization (Zamanzadeh et al., 2019). Ethical leadership (EL) influences all stakeholders of an organization, e.g., employees. That said, most researchers have focused on external stakeholders only, thereby undermining the critical role of employees (Chen and Hou, 2016; Xu et al., 2016; Hoch et al., 2018; Rodriguez, 2018; Ahmad et al., 2019). Previously, many considered EL superfluous and impractical, a concept contradicting the key perspective in organizational behavior and organizational psychology (Smith et al., 2016). Recent research, however, has succeeded in highlighting the essence and true potential of ethics in the workplace, especially EL (Javed et al., 2019).

Ethical leadership signifies a leader who respects ethical beliefs and values, prioritizes the dignity and rights of subordinates (Byun et al., 2018), and focuses on moral development and virtuous behavior (Mo and Shi, 2017). Thus, EL is often associated with concepts like trust, honesty, consideration, charisma, fairness, power-sharing, role clarification, and integrity (Lee et al., 2019b). It engenders positive outcomes, such as JE, WENG, and CS (Ling et al., 2017). In the context of employee retention and loyalty, JE is an important factor representing the collection of forces that influence employee retention (Ahn et al., 2018). The concept of JE differs from turnover in its acute emphasis on all aspects of keeping an employee on the job instead of the psychological process one goes through when quitting (Badrinarayanan et al., 2018). Extant works consider EL superior to other styles in harnessing organizational loyalty to serve beyond contractual limits (Ju et al., 2019). Ahmad and Gao (2018) assert that EL entails the potential to stimulate positive behaviors and attitudes (e.g., vigor, dedication, WENG, motivation, and psychological work ethics) among followers. A work culture in which employees relate the degree of satisfaction with work-related outcomes, achievements of certain goals, career commitment, proactive personality, diversity training, goal orientation, organizational learning, and development feedback (Pathardikar et al., 2016) can enhance employee CS and organizational commitment (Ahmad and Saad, 2020). Congruent with prior assertions, it is safe to assume that EL strengthens JE and WENG, thereby contributing to employee CS.

From an industry perspective, most industries follow a set of ethical guidelines, yet the hospitality industry has sometimes proven itself to be less than hospitable (Guzzo et al., 2020). As EL is an emerging concept in the hospitality sector with few studies, scholars (Zhang et al., 2020) have recently called for new models explaining the mediating role of JE in shaping employee career adaptability and success in the hospitality industry. Wang et al. (2019) added that investigating career skills and WENG as potential mediators could help resolve critical organizational and employee-centric issues. Drawing from the Attitude Theory (ATT) and Social Exchange Theory (SET), Kaya and Karatepe (2020) found that WENG (as a mediator) performed superior to the direct effect model when explaining the influence of servant leadership (SL) on CS and adaptive performance. In a meta-analytical study, Lee et al. (2019a) encouraged organizational and occupational psychology scholars to incorporate multiple mediators (i.e., WANG and JE) in new leadership models for better understanding. Another pressing concern in the current EL literature on the hospitality industry is the lack of longitudinal studies. At the same time, previous findings built on cross-sectional data are not generalizable, undermining the industrial tenure of employees. Kaya and Karatepe (2019) argued that longitudinal data could be more useful in predicting relationships and generalizing empirical findings to other hospitality or service settings.

The main purpose of this work is to address previous knowledge gaps by examining the direct and indirect links between EL, CS, JE, and WENG using data from hospitality workers in China. This study directly responds to Kaya and Karatepe (2019), who called for establishing the sequential mediation effect of JE and WENG as serial mediators in the relationship of EL and CS. Van Knippenberg and Sitkin (2013) also asserted the need for deepening knowledge about the multifaceted effects of EL on employee outcomes. Some unique aspects of this study are discussed hereafter. Firstly, to the best of our knowledge, this study presents the first empirical model examining the sequential mediation effect of JE and WENG between EL on CS using time-lagged data of Chinese hospitality workers. Second, in line with recent calls in the OB literature (Wang et al., 2019; Lee et al., 2020), the paper makes an initial attempt to combine three distinct theoretical paradigms—i.e., the SET, the ATT, and the Organizational Support Theory (OST)—to explain the influence of EL on CS, JE, and WENG.

The remaining part of this research study is structured as follows. The introduction is followed by section “Review of the Literature and Hypothesis Development,” which includes key concepts and a critical review of past theories and empirical studies for hypotheses development. Section “Outcomes of Ethical Leadership: A Theoretical Perspective” outlines the methodology, consisting of information on samples, instruments, and procedures. Section “Results and Findings” presents key results and analysis. Section “Discussion” includes a discussion of the main findings and their academic and managerial implications, followed by limitations and future directions. The last section is the conclusion summarizing the outcomes of this study.

## Review of the Literature and Hypothesis Development

### Key Concepts

Ethical leadership is often defined as a placid, respectful, caring, honest, and service-oriented individual with a high sense of justice community building (Lee et al., 2017; Peng and Lin, 2017). JE can be defined as the sum of three factors promoting employee retention in an enterprise. These factors are: (i) *links* representing informal and formal interactions among organizational members and employees; *fit* refers to the perceived fitness (compatibility) of a worker with community and enterprise; (ii) *sacrifice* denotes the perceived material, psychological, and social costs incurred by an employee in case of quitting an organization (Yasir and Rasli, 2018). The concept of WENG, comprises three aspects i.e., vigor, dedication, and absorption. It signifies a state of work in which the individuals employ and express themselves physically, cognitively, emotionally, and mentally while performing any task (Aryati et al., 2018). CS is a popular and frequently-researched concept in occupation, work, and retention literature, embodying all work-related activities a person engages in and all of the work-related experiences a person has throughout a lifetime (Shareef and Atan, 2019). In the modern business context, the antecedents of CS extend beyond monetary values to subjective efforts by leaders.

## Outcomes of Ethical Leadership: A Theoretical Perspective

Ethical leadership is often described as “the demonstration of normatively appropriate conduct through personal actions and interpersonal relationships and the promotion of such conduct through two-way communication” (Brown and Treviño, 2005, p. 120). Extending beyond the typical normative and philosophical description of EL, this definition emphasizes consequences and behaviors: (i) establishing credibility through the enactment of ethical and normative perspective; (ii) paying attention to ethical dimensions raised by members and organization; (iii) openly discussing ethical issues through two-way communication; (iv) creating fairness-based procedures and relationships; (v) rewarding compliance with ethical rules, standards, and norms (Brown and Treviño, 2005, p. 120); (vi) intervening on followers failing to comply with ethical norms (Treviño et al., 2003; Benevene et al., 2018). More importantly, the latter part of the definition delineates a decision-making process, highlighting that organizational members may emulate the behaviors and choices of leaders. EL should be fair when making decisions and keep themselves aware of possible consequences of their decisions in light of what they promote and enact (Benevene et al., 2018).

The theoretical underpinning of EL and its potential impact on various organizational outcomes can be interpreted through the lens of multiple theories: SET (Blau, 1964), ATT (Breckler and Wiggins, 1989), and the OST (Eisenberger et al., 1986). Extant research offers three underlying mechanisms to explain potential antecedents and consequences of CS. Firstly, SET (Blau, 1964) proposes that an exchange-oriented relationship governs transactions among organizational members. Employees and employers follow certain rules, standards, norms, and social values and share resources in a win-win scenario. This reciprocal relationship involves a set of beliefs, expectations, reciprocity, caring for individual and organizational wellbeing, and following rules, policies, strategies, and obligations (Ampofo, 2020). Secondly, the ATT (Breckler and Wiggins, 1989) posits that individual behaviors—shaped by certain attitudes experienced in any event, object, person, or situation—are the basis of their actions and reactions (Zhang and Zhang, 2020). Attitude is an individual’s psychological tendency toward particular social things. Since all employees espouse certain attitudes toward their superior, compliance of leaders with ethical practices serves as a key driver in establishing a positive environmental climate. Employees respond by showing loyalty, dedication, compassion, engagement, and gratification (Nolder and Kadous, 2018).

Thirdly, OST (Eisenberger et al., 1986) is another important concept delineating that employee response to an organization depends on the degree to which they believe that their organization values their contributions, cares about their wellbeing, and fulfills socio-emotional needs. Employees perceiving strong organizational support would identify their leader as ethical (Ahmad et al., 2019). Drawing from the above three concepts, it is rational to assume that followers of ethical leaders are more likely to form a positive perception and attitude about their leader based on past experiences, e.g., honesty, integrity in social exchanges, fair judgments, and moral stance. If employees believe that their leader is making all possible efforts to ensure that all employees, irrespective of their background, receive the needed organizational support, they will be more embedded in their job and utilize all available opportunities to engage, eventually feeling more satisfied with their career progress.

### Ethical Leadership and Career Satisfaction

Past evidence validates that individuals’ ethical beliefs often influence their behaviors to an extraordinary degree of engagement and satisfaction (Ahmad et al., 2019). A study demonstrated that the ethical fit between employee and employer in the African insurance industry ended in high commitment, devotion, and loyalty (Pathardikar et al., 2016). Employees perceiving these factors have a high sense of CS as they work selflessly for their organizations. Afsar et al. (2018) reported that the fairness perception of ethical leaders enables them to create strong psychological contracts with employees. In another study, Ahmad et al. (2019) analyzed employee and employer dyadic data in an international fast-moving consumer goods company in Pakistan. The authors confirmed that EL facilitated positive employee perception of job security and CS by lifting employee morality. Another study by Chughtai (2018) in the food and beverage industry showed that EL causes CS. EL, acting as passive leaders, could help favorably promote CS (cf. see for review of passive leadership, Jones and Jones, 2017). In Turkey, a study documented employee response to EL in private companies. The results verified that EL incites employee creativity and satisfaction (Karabey and Aliogullari, 2018). A recent Chinese study affirmed that EL significantly enhances career calling (Zhang and Jiang, 2020). Thus, it is rational to speculate that the positive influence of EL on CS originates from a social exchange process, where leaders institutionalize and exemplify high ethical protocols, enabling employees to espouse favorable dispositions about their CS. In line with the above, the following hypothesis is proposed:

**H_1_:** EL positively affects CS.

### Ethical Leadership, Job Embeddedness, and Career Satisfaction

Many authors have noted that EL espouses integrity and fairness by practicing transparent modes of ethics. Such practices nurture identity, motivation, JE, leader-member exchange, psychological contract, and organizational engagement (Chen and Hou, 2016; Xu et al., 2016; Hoch et al., 2018; Rodriguez, 2018; Ahmad et al., 2019). Several preceding studies have validated positive links between EL and JE in different forms (Lee and Huang, 2019). In another study, Al-Ghazali (2020) built an OST-based model to examine the nexus between transformational leaders, career success, and CS using data from nurses working in the government hospitals in Saudi Arabia. The data affirmed that leadership enriches perceived career success leading to CS. Another United States study empirically established JE as an antecedent (and a mediator) to CS (Huning et al., 2020). An empirical investigation of employees in a 5-star hotel in Egypt revealed that inclusive leadership promotes JE (Elsaied, 2020). Another study on flight attendants in Turkey indicated SL as a predictor of JE and CS (Ilkhanizadeh and Karatepe, 2018). Likewise, Linder (2019) observed that positive organizational embeddedness contributed to CS among managers and expatriates. In another study, Ferreira et al. (2017) validated that JE (as a mediator) explains the relationship between the leader’s ethical foundations and CS. The authors gathered employee responses from 46 hotels in Portugal. For China, another study found that leaders and followers of media organizations who experienced ethical spectrums in the early stage of their career demonstrated strong JE traits (Chen and Hou, 2016). Based on the above, the current study builds on tenets of the ATT and SET to propose the following hypotheses:

**H_2_:** EL positively affects JE.

**H_3_:** JE positively affects CS.

### Ethical Leadership, Work Engagement, and Career Satisfaction

There is considerable evidence supporting that EL can effectively mobilize employees for WENG (Engelbrecht et al., 2017). An ethical climate created through efficient leader-member exchange can pave the way for overall employee satisfaction in all aspects, especially career progression (Rafiq, 2019). A few studies have examined WENG and career aspirations as antecedents to EL (Guarnaccia et al., 2018). To offer meaningful contributions, EL strives hard to care for employee emotions and sentiments that keep them in an ongoing WENG phase, enabling robust social and trusting exchanges, leading to superior outcomes (Yang and Wei, 2017). Ahmad and Gao (2018) found that EL working in the banking sector successfully incited WENG to yield favorable outcomes. Likewise, Amos et al. (2017) observed that employees of an electronic web-based company reported trust and WENG for leaders displaying integrity and ethics. In another phenomenological study, Özsungur (2020) found that employees working at the Turkish Chamber of Commerce linked EL to WENG and career success. In Korea, followers of EL perceived engaged in their work, satisfied with their careers, and felt a greater sense of well-being in their lives (Baek-Kyoo and Insuk, 2017). Another study on Australian teachers concluded that WENG generates CS among employees, especially when the work environment is built on ethical practices (Emmerich and Rigotti, 2017). Thus, it is predicted that:

**H_4_:** EL positively affects WENG.

**H_5_:** WENG positively affects CS.

### Job Embeddedness and Work Engagement

Past theory and research provide sufficient evidence to predict that employees who are more embedded in their jobs pursue WENG to realize their individual and organizational goals (Yozgat and Meşekıran, 2016). The positive impact of quality bonding between EL and followers has been frequently tested and validated in the past literature (Dan et al., 2018). Munsir and Irwandy (2020) investigated the WENG-JE nexus at the RSUD Haji of South Sulawesi Province. They found a significant influence of JE on WENG, a view consistent with a later study on employees of a luxury hotel (Yu et al., 2020b). In another study, the investigators gathered opinions of graduate students majoring in hotel management. The interviewees considered JE a critical phase in establishing WENG (Koo and Lee, 2020). Engelbrecht (2020) explains that the holistic notion of fit, link, and sacrifice is closely linked to WENG, though ethical values serve as the driving force. Of significance, a few earlier employee-leadership surveys have also reported positive interactions between JE and WENG in the Ghanian hospitality industry (Ampofo, 2020) and the tourism sector of China (Yu et al., 2020a). Based on the above empirical and conceptual evidence, it is hypothesized that:

**H_6_:** JE positively affects WENG.

### The Mediating Role of Job Embeddedness and Work Engagement

Ethical leadership can shape employee attitudes and behaviors toward JE by ensuring that all employees get equal opportunities, employee efforts are valued, all available resources are deployed to retain them and make their work environment more inclusive (Zhang et al., 2020). Employees work more passionately when they perceive a trusting bond and strong relationship with their managers or leaders (Chin et al., 2016). Kaya and Karatepe (2020), among other authors, believes that the mediating influence of JE is one of the potential factors that could help unwrap the precise mechanism through which EL generates positive outcomes, e.g., CS. Kim (2019) added that EL actively seeks opportunities to keep employees satisfied with their work and careers. They eradicate potential threats to employee embeddedness by setting clear and transparent mechanisms in all aspects of work (e.g., punishments, rewards, and resources) and create conducive relationships based on trust, care, and support, while institutionalizing ethical agenda. Nonetheless, many scholars have shown significant interest in explaining the role of JE (Lee et al., 2019a) and WENG (Ahmad and Gao, 2018) as potential mediators between EL and CS.

Explaining the sequential mediation perspective, Al-Ghazali (2020) stated EL might be indirectly related to WENG through JE. Even though JE is a strong predictor of CS when considering EL, integrating multiple mediators (i.e., WENG and JE) in the same equation could bring new insight (Kaya and Karatepe, 2020). Lyu and Zhu (2019) further demystified the sequential scheme of mediators by proposing that the impact of EL on WENG might not be straightforward. One possible mediating mechanism is JE. Employees experiencing quality contracts, relationships, and exchanges with their leaders are expected to have a higher level of CS, provided that they are embedded and engaged at work under ethical principles (Grobelna, 2019). Based on the preceding arguments, the following hypotheses are speculated:

**H_7_:** JE mediates the relationship between EL and CS.

**H_8_:** WENG mediates the relationship between EL and CS.

**H_9_:** JE and WENG sequentially mediate the relationship between EL and CS.

**FIGURE 1:**
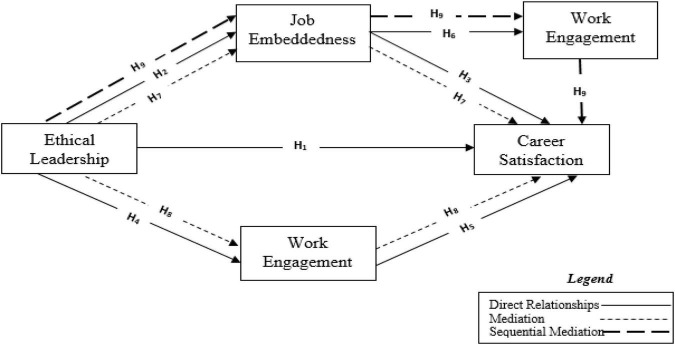
The conceptual framework.

Below, Figure 1 shows the proposed conceptual model, depicting the direct and indirect relationship between EL, CS, JE, and WENG. The model accounts for the parallel and sequential mediation effects of JE and WENG between EL and JE.

## Methods and Measures

### Sample and Procedures

Primary data were collected through a survey questionnaire from employees of four hotels (two 5-star and two 4-star) in Xiamen City, China, with a total population (*N* = 1189; hotel employees). Data collection followed a simple random sampling technique. After contacting seven hotels in the twin cities, the management of only four hotels granted permission for data collection. Self-completion questionnaires were disseminated among more than 350 employees, including senior and junior management staff, reception clerks, guest relations representatives, waiters and waitresses, and concierges who had frequent interactions with customers. Data were collected in three-wave, i.e., each survey was conducted with a gap of almost 30 days. The first phase (Time 1) resulted in 311 submissions, out of which three were excluded due to incomplete and improper responses. The remaining 308 questionnaires were selected for the second phase (Time 2), resulting in 289 responses and eleven omissions. Out of 278, 256 usable questionnaires were collected in the final phase (Time 3) and nine incomplete questionnaires. A total of 247 responses were kept aside for final data analysis, yielding a response rate of 79.4%. The homogeneous unit of analysis could potentially contaminate or inflate the relationships among variables due to common method variance. Therefore, Harman’s single-factor test was utilized to check if unaccounted variance by a single factor. The test score (cumulating variance = 23 percent) showed that the bias was not a serious threat to the current study. IBM SPSS 21 and IBM SmartPLS3 were used for data analyses.

### Measures

This study used well-established and reliable instruments for measuring variables adopted from previous studies. The number of items, measurement time, and sources are as follows: EL-ten items, Time 1, Brown and Treviño (2005); JE-seven items, Time 2, Crossley et al. (2007); WENG-nine items, Time 2, Schaufeli et al. (2006), with three items for each dimension, i.e., vigor (VG), dedication (DD), and absorption (AP); CS-five items, Time 3, Greenhaus et al. (1990). All items were measured on a 5-point Likert scale, where 1 = *strongly disagree* to 5 = *strongly agree*.

## Results and Findings

Annexure 1 presents the summary of descriptive statistics. Table 1 summarizes the reliability and validity statistics of the measurement model (Fornell and Larcker, 1981). As per commonly accepted standards (Hair et al., 2016), the reliability and validity statistics (convergent and discriminant) were used as criteria for assessing the model measurement. PLS algorithms were run to calculate the outer loadings for all constructs. As seen in Annexure 2, all factor loading scores were well above the prescribed threshold of 0.7, demonstrating significant interaction among the factors and the variables. The average variance extracted (AVE) values for all constructs were greater than 0.5 on average, showing statistically acceptable convergent validity (see for review, Henseler et al., 2009). Next, a redundancy analysis was carried out to gauge the reliability and validity of WENG (a second-order construct). Table 2 shows the summary of the redundancy analysis, depicting that all WENG dimensions (VG, DD, and AP) had significant weight over their respective variable.

**TABLE 1:** Reliability and convergent validity.

Constructs	Cronbach’s Alpha	Composite Reliability	(AVE)
EL	0.918	0.933	0.584
JE	0.828	0.871	0.593
CS	0.812	0.870	0.550
VG	0.889	0.931	0.818
DD	0.869	0.920	0.792
AP	0.870	0.920	0.793

*EL, ethical leadership; CS, career satisfaction; JE, job embeddedness; WENG, work engagement; VG, vigor; DD, dedication; AP, absorption.*

**TABLE 2:** Redundancy analysis: weight and significance of dimensions.

WENG dimensions	Weight	Sig
VG	0.367	20.347
DD	0.399	29.121
AP	0.383	29.542

*VG, vigor; DD, dedication; AP, absorption.*

To check the model fitness, the SRMR (Standardized Root Mean Square Residual) value less than 0.10 or 0.08 (in a more conservative version) is considered a good fit (Hu and Bentler, 1999). The SRMR value was well above the acceptable standards, i.e., 0.023. Henseler et al. (2014) described the SRMR as a “goodness of fit measure” for PLS-SEM that can be used to avoid model misspecification. As per standard (NFI > 0.9), the NFI (Normed Fixed Index) of the model (0.988) also represented a good fit.

Table 3 depicts the summary of results of the HTMT ratio of correlations to measure the discriminant validity of latent constructs. Henseler et al. (2015) consider this method a more accurate measure of validity than previous methods. As seen below, the HTMT values of all constructs were below the acceptable threshold, i.e., > 0.9 (Henseler et al., 2015).

**TABLE 3:** Summary of results of the HTMT ratio of correlations.

Constructs	AP	CS	DD	EL	JE	VG	WE
AP							
CS	0.885						
DD	0.805	0.873					
EL	0.631	0.714	0.684				
JE	0.688	0.733	0.736	0.865			
VG	0.555	0.723	0.687	0.708	0.765		
WENG	0.979	0.832	1.029	0.762	0.824	0.894	

*EL, ethical leadership; CS, career satisfaction; JE, job embeddedness; WENG, work engagement; VG, vigor; DD, dedication; AP, absorption.*

**FIGURE 2:**
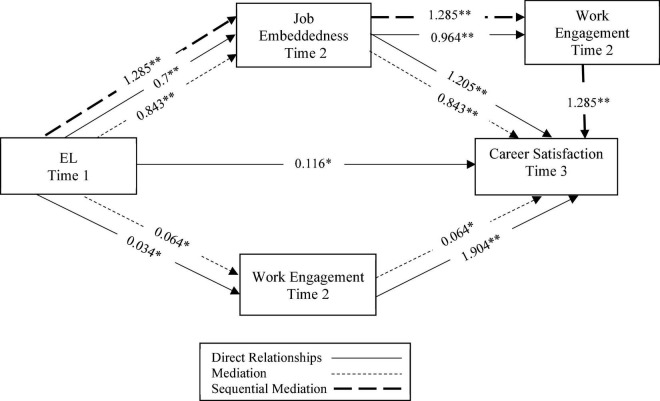
Summary of results of the structural model with direct paths, mediation, and sequential mediation effects. *Correlation is significant at 0.05 (2-tailed), **Correlation is significant at 0.01 (2-tailed).

Table 4 outlines the summary of results of the hypotheses testing, comprising the direct, indirect, and mediation effects in the structured model (see also, Figure 2). The present study evaluated a structural model to investigate the link between EL and CS through the sequential mediating effects of JE and WE. Some of the key findings for the direct effects are discussed hereafter. First, the *R*^2^ value showed that all indicators predicted 67.5 percent of CS. Second, the outcomes indicated a significant and positive relationship between EL and CS (β = 0.116, *p* < 0.05), confirming that H_1_ was accepted. This result validated that the implementation of EL in the hospitality industry can yield higher CS. Third, the results revealed a significant and positive impact of EL on JE (β = 0.700, *p* < 0.01), thereby affirming the acceptance of H_2_. This result implied that EL contributes to a high sense of JE among followers. Fourth, the data analysis confirmed the acceptance of H_3_, as evident by the effect of JE on CS (β = 1.205, *p* < 0.01). This outcome empirically validated JE as a strong tool to increase CS. Fifth, EL emerged as a significant predictor of WENG among followers (β = 0.034, *p* < 0.05), augmenting the acceptance of H_4_. Sixth, WENG positively incited CS (β = 1.904, *p* < 0.01), implying that engaged employees are more satisfied with their careers. Hence, H_5_ was accepted. Seventh, the empirical estimates supported the positive influence of JE on WENG, approving H_6_.

**TABLE 4:** Summary of the results of the structured model: direct and indirect effects.

Hypotheses	β-values	t-statistics	*p*-values	f^2^	5.00%	95.00%	Result
H_1:_ EL → CS	0.116	1.935	0.027	0.421	0.009	0.208	Supported
H_2:_ EL → JE	0.700	19.814	0.000	0.960	0.624	0.745	Supported
H_3:_ JE → CS	1.205	3.763	0.000	1.205	1.76	0.728	Supported
H_4:_ EL → WENG	0.034	1.846	0.033	0.325	0.005	0.065	Supported
H_5:_ WENG → CS	1.904	6.068	0.000	0.244	1.363	2.43	Supported
H_6:_ JE → WENG	0.964	61.586	0.000	0.107	0.937	0.988	Supported
H_7:_ EL → JE → CS	0.843	3.559	0.000	–	1.27	0.473	Supported
H_8:_ EL → WENG → CS	0.064	2.269	0.012	–	0.023	0.113	Supported
H_9:_ EL → JE → WENG → CS	1.285	5.258	0.000	–	0.891	1.728	Supported

For the mediation effect, the results of the mediation analysis using the bootstrapping technique provided empirical bases for the acceptance of the partial mediation role of JE (H_7:_ β = 0.843, *p* < 0.01) and WENG (H_8_: β = 0.064) between EL and CS, but the latter was only significant at *p* < 0.05. Apart from supporting H_9_, the outcomes of sequential mediation (β = 1.285, *p* < 0.01) showed that EL successfully incites a sense of CS among followers by first uplifting JE that enhances WENG, causing CS. Effect sizes (f^2^) in this study were examined using standards outlined in the work of Gefen et al. (2011). Effect size determines the impact of exogenous latent constructs on endogenous variables. The current study noted most of the values with larger effect sizes. It means that the overall impact of EL on the other constructs is remarkable. The f^2^ values larger than 0.35 denote a larger effect size, 0.15 medium effect side, and 0.02 donates a small effect size.

## Discussion

This study has made an initial attempt to unwarp the complex underlying factors through which EL prototypes enable employees to feel more satisfied with their careers. For this purpose, a structured model was constructed with multiple factors, including EL (antecedent), CS (outcome), JE (Mediator 1), and WENG (Mediator 2) to examine potential direct and indirect effects, particularly the sequential mediation effect of mediators in response to recent academic calls (cf. Kaya and Karatepe, 2020, see also Al-Ghazali, 2020). The paper used a robust approach to collect views of 247 hospitality workers in China in three phases for different study variables. The data analysis through SPSS and SmartPLS3 revealed the following results. Firstly, corroborating past views (e.g., Chughtai, 2018; Karabey and Aliogullari, 2018; Ahmad et al., 2019), the empirical estimates suggested that hotel employees perceive more satisfaction with their careers when leaders espouse integrity, fairness, social motivation, honesty, trustworthiness, and champions mutual dialogue and empowerment in social exchanges and organizational support mechanisms.

Secondly, the current empirical assessment suggested that senior management, managers, or supervisors involved in ethical perspectives are more effective in activating JE among hospitality workers. They do so by keeping a close eye on employees’ cognitive dispositions concerning fit, links, and sacrifice. The results suggest that EL develops a conducive environment and establish transparent and inclusive links among employees and key stakeholders (e.g., community and organization) to ensure that employees feel relevant and valued, thereby paving the way for pro-leaders and organization-centric sentiments. This finding makes sense, given that employee assessment and response to leaders and organizations depend on the perceived emotional, financial, and ethical support (Lee and Huang, 2019). In line with earlier assertions (cf. Kim, 2019; Lee et al., 2019a), the enactment, personification, enactment, and promotion of high morals and ethics encourage followers to be more embedded in their work, remain satisfied and resent leaving their organization considering the material, psychological, and social sacrifices they may incur. More so, the empirical model explains that dissemination of ethical values and climate by leaders provides a cognitive basis to stay embedded and develop behaviors and attitudes that translate into satisfaction and commitment (do Nascimento et al., 2018). Specifically, the current data analysis demonstrated that JE, as a vital antecedent to CS (Linder, 2019), also complements the effect of EL on CS.

Thirdly, adding credence to the seminal works of Brown and Treviño (2005) and later studies (Van Knippenberg and Sitkin, 2013; Benevene et al., 2018), the current findings demonstrated that leaders displaying ethical norms in personal undertakings, interpersonal exchanges, and organizational practices enhance employee engagement at work. Moreover, the present analysis, consistent with prior beliefs (Emmerich and Rigotti, 2017; Yang and Wei, 2017; Özsungur, 2020), revealed that WENG contributes to employee satisfaction and serves as an important dimension explaining the positive influence of EL on CS.

Fourthly, a new insight emerging from this work is that a cognitive schema predicts employees’ satisfaction under an EL. As predicted by earlier authors (Lyu and Zhu, 2019; Al-Ghazali, 2020; Kaya and Karatepe, 2020), empirical evidence showed that ethical deeds and actions of leaders nurture favorable attitudes and behaviors toward organizational aspects (e.g., leadership practices, quality of exchanges, and support) among followers and keep them embedded in their work (i.e., fit, links, sacrifice). This state of mind cultivates loyalty, commitment, and engagement to leaders and work context, eventually translating to happy and satisfied employees.

## Theoretical Implications

From a theoretical perspective, the current findings add to the current knowledge and research on the various effects of EL, especially in an underresearched organizational context, i.e., the hospitality sector. The study, built on the tenets of SET, ATT, and OST, tested its direct and indirect effects on employee outcomes, namely JE, WENG, and CS, demonstrating the significance and relevance of these relationships. Of important theoretical implications, the paper extends the knowledge on the mediating factors complementing the influence of EL, an area lacking empirical studies (Van Knippenberg and Sitkin, 2013; Benevene et al., 2018; Kaya and Karatepe, 2020). Past works in the EL literature have explored various mediating factors, including work meaningfulness, psychological capital, LMX, ethical climate, and trust (Benevene et al., 2018). Yet, the precise sequential process of JE and WENG in between EL on CS has not been researched, particularly in the context of the hospitality industry.

In the same vein, the study expands the literature built on the SET (Brown and Treviño, 2005; Van Knippenberg and Sitkin, 2013; Benevene et al., 2018), suggesting that EL holds the potential to incite satisfaction among workers (Benevene et al., 2018). Previously, studies have proposed that employees’ satisfaction levels and reactions are contingent on their cognitive evaluations of leaders and various aspects of their work-life (Treviño et al., 2003; Pettijohn et al., 2008; Benevene et al., 2018). Yet, the present results provide one feasible explanation to demystify the influence process. Employees develop positive attitudes, a sense of pride, and altruism in response to an ethical environment where ethical leaders and followers comply with high ethical standards, exchange, transact freely and fairly and care for each other’s needs and goals. Employees respond to this inclusive exchange culture by demonstrating increased embeddedness and engagement in their work, with an increased level of satisfaction. Colloquially, the present outcomes also resonate with the Attachment Theory, positing that employee satisfaction upsurges in context with solid relational relationships (Haller et al., 2018). For hospitality workers constantly facing challenging and dynamic environments, especially in the current times, EL can provide safe working conditions for employees, strong dyadic feedback mechanisms, reduce anxiety, and mitigate work-load, often associated with turnover and satisfaction (Qin et al., 2014). As of this date, only a few studies have highlighted this aspect of EL (e.g., Benevene et al., 2018).

## Practical Implications

The outcomes of this study offer some practical suggestions for how hotels can effectively address issues in employee management. As observed, the emergence of EL as an effective antecedent to employee perception of CS calls for the integration of ethical dimensions in all aspects of hotels management and operations, including but not limited to training, selection, and recruitment. Team leaders and other management leaders should be trained and selected based on their ability to diagnose and prevent indicators adversely influencing the ethical climate and satisfaction. Congruent with the above, hotel management should empower leaders with all resources, allowing them to be consistent with the ethical values they enact and promote, thereby enabling employee trust in leaders and emulation of ethical norms, rules, procedures, and practices among team members.

Furthermore, the present findings imply that hotels should encourage managers and supervisors to enact EL to improve work-centric embeddedness among employees. These ethical qualities and behaviors should be targeted at improving three critical aspects, i.e., links, fit, and sacrifice. For links, managers should focus on: (i) building cohesive relationships among employees through social events, birthdays, and other celebrations; (ii) cultivating trust through integrity, honesty, fairness, team-building activities, coaching, and open dialogue; leading by example; (iii) establishing links with leaders and members; discussing problems to seek shared solutions. Managers can address employee fit perception by ensuring that employees are in a role that fits their skillset and is perceived as meaningful. They should align individual and team goals with the organization, so employees see value and meaning in their work to contribute to the greater good. They should individually consult employees about their careers and help them create and stick to a career plan. Managers should assist employees in crafting and defining their values while inspiring them to emulate ethical values by acting as role models. They should also educate employees on linking and using personal and ethical values with tasks and activities. Moreover, in line with the attributes of EL, management should focus on creating leaders who constantly strive for a healthy, happy, comfortable, safe, and stress-free working atmosphere. If employees are appreciated for their work through rewards, praises, and kind gestures, they are less likely to sacrifice their job. As empirically demonstrated in this study, employees are more likely to identify with leaders’ values and emulate them in their work in response to the above initiatives. In an attempt to reciprocate, employees would consider it a moral obligation to engage whole-heartedly, feeling satisfied with their job and progressing career.

Of significance to the current context, the present findings set the foundation for revisiting management and leaders in Chinese hotels, paying more attention to physical necessities, and undermining workers’ ethical, moral, and psychological requirements. The inclusion of the EL into the leadership inventory across the hospitality industry may address reported employee issues (e.g., theft, hotel espionage, whistleblowing, job switch, and poor implementation of codes of ethics) (Guzzo et al., 2020). EL can also help employees become more ethical, embedded, happy, and engaged in their work, improving their overall productivity, attitudes, and behavior to meet the demands of the growing hospitality industry.

## Limitations and Future Research

This study has some limitations that offer opportunities for further investigation. First, this study is limited in terms of geographical and industry contexts. Researchers are expected to cover more sectors and cultures to implement applications of current findings in a broader spectrum. More so, the conceptual model should be tested in non-commercial settings (i.e., the not-for-profit sector) facing high turnover. Second, time-lagged data were collected over three measurement points. Measurement with more time lags is expected to bring new insight. Third, this paper may be considered a census study, and the findings are set to be generalized for the concerned population only [i.e., four hotels (two 5-star and two 4-star) in China. Future research should consider collecting data from small hotels with turnover ratios (Park and Min, 2020). Fourth, this paper focused on three underlying mechanisms, i.e., JE and WENG. Academics (e.g., Khan, 2020) have proposed other variables (e.g., psychological capital, psychological safety, psychological contract fulfillment, psychological safety, or psychological attachment) as moderators for a moderated mediation model to analyze the objective measures of organizational support] as crucial mediating mechanisms explaining different outcomes of leadership (cf. Elsaied, 2020). Thus, the model should be tested with more variables and leadership styles (e.g., authentic) in moderation-mediation models. Fifth, the study used three academic paradigms (i.e., ATT, SET, and OST) to explain the relationship between EL, CS, JE, and WENG. Using an alternate theoretical basis (e.g., the Attachment Theory), in conjunction with the current framework or separately is encouraged.

## Conclusion

The present paper has attempted to address the theoretical and empirical gaps in the existing literature on factors affecting employee engagement by demonstrating the sequential mediation mechanisms of JE and WENG, enabling EL to improve CS among hospitality workers in China. The current empirical results confirm that EL significantly contributes to the professional commitment of hospitality workers as they reciprocate ethical norms and values of leaders with increased JE, which in turn inspires them to engage in work, making them more satisfied with their careers. More so, the mediation effects suggest that employees’ sensitivity attitude toward ethical leaders’ practices, including the value of dyadic interactions or exchanges, fairness, integrity, rewarding attitude, procedural cleverness in people management, cognitive and physical alignment of workers in well-suited roles enables them to see themselves engaged in a meaningful pursuit for career progression. The findings suggest that, among other potential factors, hotel management must recognize the importance of this cognitive mechanism stated above through effective changes in human resources practices to address employee concerns and improve their ethical undertakings, commitment, loyalty, satisfaction, and productivity.

## Data Availability Statement

The original contributions presented in the study are included in the article/supplementary material, further inquiries can be directed to the corresponding author.

## Ethics Statement

The studies involving human participants were reviewed and approved by the Jimei University, China. The patients/participants provided their written informed consent to participate in this study.

## Author Contributions

AH and SK: conceptualization. SA and SR: methodology. LS, AH, and SK: software. SA: validation. SR: formal analysis. LS: investigation. SK and SA: resources. AH: data curation. SR, AH, and LS: writing original draft preparation. SK, SA, and SR: writing, review and editing. SK and LS: supervision. AH and LS: project administration. SA and LS: funding acquisition. All authors have read and agreed to the published version of the manuscript.

## Conflict of Interest

The authors declare that the research was conducted in the absence of any commercial or financial relationships that could be construed as a potential conflict of interest.

## Publisher’s Note

All claims expressed in this article are solely those of the authors and do not necessarily represent those of their affiliated organizations, or those of the publisher, the editors and the reviewers. Any product that may be evaluated in this article, or claim that may be made by its manufacturer, is not guaranteed or endorsed by the publisher.

## Figures and Tables

**Annexures II:**
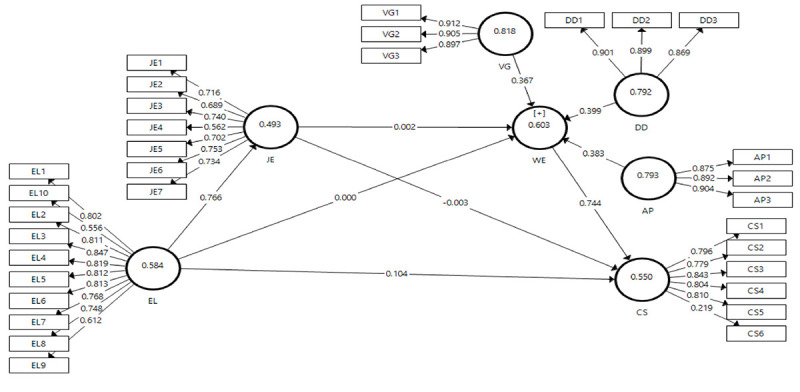
Outer loading scores.

## Annexures

**Annexures I:** Summary of descriptive statistics.

Demographics	Codes	Frequency	Percentage (%)
Gender	(1) Male	139	57
	(2) Female	108	43
Age	(1) 25 and Less	31	12.5
	(2) 26–30	46	18.6
	(3) 31–35	53	21.5
	(4) 36–40	79	31.9
	(5) Above than 40	38	15.4
Hotels participants	(1) Hotel 1: 4-star	64	25.9
	(2) Hotel 2: 4-star	85	34.4
	(3) Hotel 3 5-star	57	23.1
	(4) Hotel 4: 5-star	41	16.6
Job Period	(1) 1 year and less	37	14.9
	(2) 1–5 years	56	22.7
	(3) 5–10 years	83	33.6
	(4) More than 30 years	71	28.7
